# A clinical and head CT-based scoring system for predicting high cerebral microbleeds burden in hypertensive patients

**DOI:** 10.1186/s12880-026-02576-w

**Published:** 2026-07-14

**Authors:** Xinbin Wang, Yonggang Qiu, Qinbin Wang, Hao Dong, Zhihua Xu, Dihong Chen, Deyun Huang, Sheng Chen, Yicheng Hu, Zebin Yang

**Affiliations:** 1 Department of Radiology, The First People’s Hospital of Xiaoshan District, Xiaoshan Affiliated Hospital of Wenzhou Medical University, Hangzhou, Zhejiang China; 2 Department of Public Health, Community Health Service Center of Guali, Hangzhou, Zhejiang China; 3 Department of Radiology, Tongde Hospital of Zhejiang Province, Hangzhou, China; 4 Department of Cardiology, Xiaoshan Affiliated Hospital of Wenzhou Medical University, The First People’s Hospital of Xiaoshan District, Hangzhou, China; 5 Department of Radiology, The Affiliated Dongyang Hospital of Wenzhou Medical University, Dongyang, China

**Keywords:** Hypertension, Scoring system, Cerebral microbleeds, Computed tomography

## Abstract

**Objective:**

This study aimed to establish and validate a scoring system utilizing clinical and head CT features to predict high cerebral microbleeds (CMBs) burden (> 10 CMBs) in hypertensive patients.

**Methods:**

A retrospective review was conducted on 458 hypertensive patients from two centers, including 244 patients in the training cohort, 101 patients in the internal validation cohort, and 113 patients in the external validation cohort. Clinical data, as well as head CT and MRI findings, were collected. Head MRI results were used to classify patients into two groups: those with > 10 CMBs and those with ≤ 10 CMBs. Statistically significant clinical and CT features distinguishing the groups were identified through univariate and multivariate logistic regression analyses. These features were subsequently weighted and scored to establish a scoring system. The model’s effectiveness was assessed through receiver operating characteristic (ROC) curves and decision curve analysis (DCA). To enhance clinical applicability, the scoring system was stratified into three score ranges.

**Results:**

Four features were ultimately incorporated into the scoring system: hypertension duration, lacunar infarcts count grade, lacunar infarcts location grade, and leukoaraiosis grade. The area under the curve (AUC) values for the training, internal validation, and external validation cohorts were 0.898 (95% confidence interval [CI]: 0.857–0.938), 0.840 (95% CI: 0.760–0.920), and 0.810 (95% CI: 0.725–0.877), respectively. At a cutoff value of 4.5 points, the sensitivity and specificity were 85.7% and 76.9%, respectively. The DCA further demonstrated the clinical utility of the model. The scoring system was categorized into three ranges: 0–1, 2–4, and 5–12. As the score increased, the incidence of high CMBs burden (> 10 CMBs) in the training, internal validation, and external validation cohorts progressively increased.

**Conclusion:**

The scoring system, which incorporates clinical and head CT features, has proven to be a valuable tool for predicting high CMBs burden in hypertensive patients and provides significant support for clinical decision-making.

## Introduction

Cerebral microbleeds (CMBs) are pathological changes caused by the rupture or leakage of intracranial microvessels, leading to the deposition of hemosiderin in the surrounding brain tissue. On T2*-weighted gradient-recalled echo (GRE) or susceptibility-weighted imaging (SWI), CMBs appear as small (2–10 mm), hypointense (black), ovoid, or rounded regions [1]. CMBs are commonly observed in individuals with hypertension and cerebral amyloid angiopathy (CAA) [2–4]. Furthermore, CMBs have been shown to be significantly associated with symptomatic intracranial hemorrhage (sICH) [5, 6]. Previous studies have defined high CMBs burden as > 10 CMBs, which is associated with a markedly increased risk of sICH following anticoagulation or thrombolysis, as well as a higher mortality rate after thrombolytic therapy [7–10]. Consequently, individualized treatment strategies are recommended for these patients [7, 9, 10]. However, current guidelines do not recommend routine magnetic resonance imaging (MRI) screening for CMBs [11, 12]. In addition, certain populations, such as patients with a cardiac pacemaker or those with claustrophobia, are unable to undergo MRI examinations. Moreover, the guidelines also advise against routine head MRI scans for acute ischemic stroke patients prior to intravenous thrombolysis, as this could lead to delays in thrombolytic treatment [11]. By contrast, head computed tomography (CT) scanning is a standard examination prior to thrombolytic therapy due to its rapid acquisition and widespread availability [13]. Therefore, the ability to predict high CMBs burden in patients about to receive anticoagulation or thrombolytic treatment using head CT scans holds significant clinical value.

Previous studies have explored the prediction of a high burden of CMBs [14, 15]. However, the prevalence of individuals with > 10 CMBs in the general population remains relatively low, ranging from 0.6% to 2.7% [11]. Therefore, focusing research on populations at high risk for CMBs is of greater clinical relevance. Notably, CMBs are strongly associated with hypertension, which itself is highly prevalent in individuals with transient ischemic attack, acute ischemic stroke (AIS), or atrial fibrillation [16, 17]. Consequently, selecting a hypertensive population as the study cohort for predicting high CMBs burden is more consistent with the clinical context.

Scoring systems are user-friendly tools and have been widely applied in clinical studies, yielding favorable results [18–20]. Therefore, this study focused on hypertensive patients and integrated clinical and head CT features to predict high CMBs burden, ultimately to develop and validate a practical and convenient scoring system for clinical application.

## Materials and methods

### Patients

**Fig. 1:**
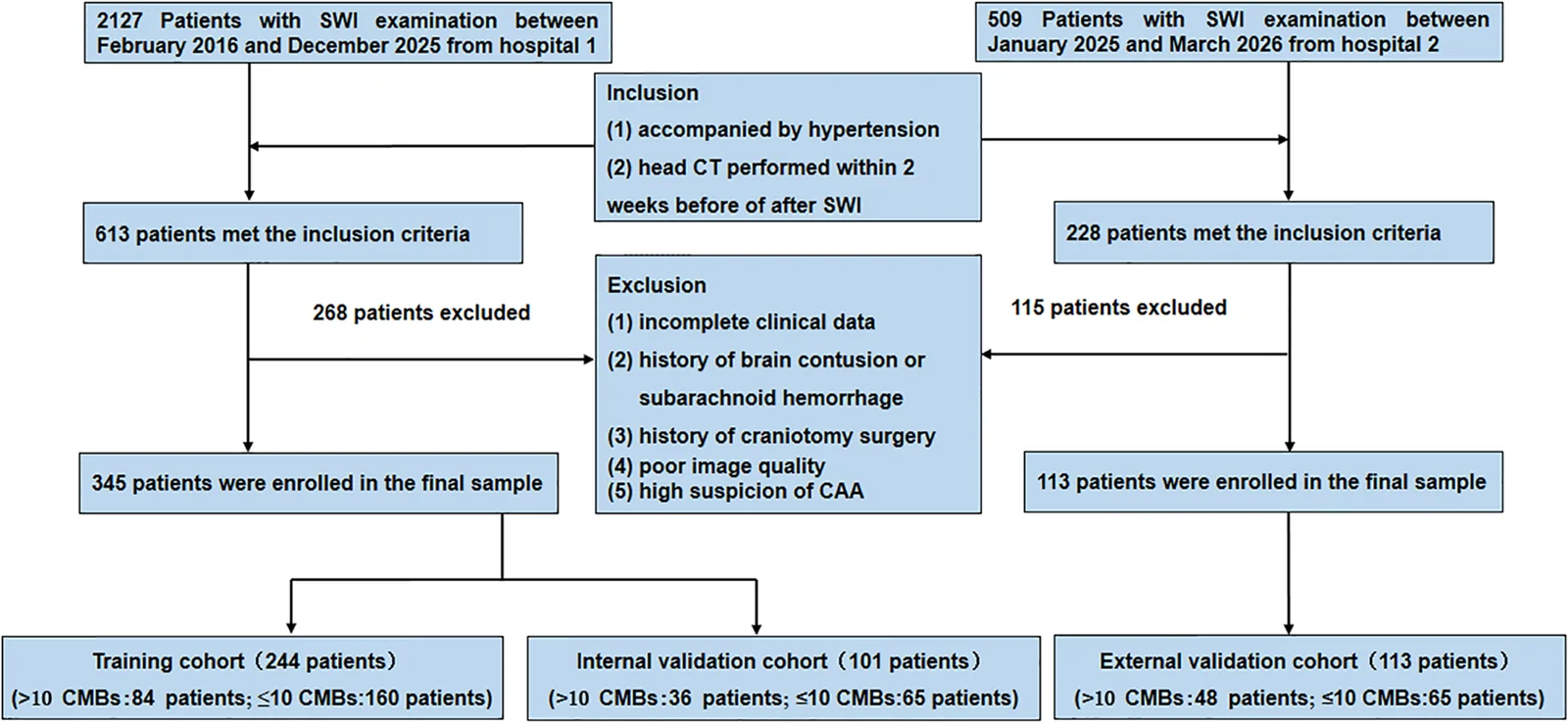
Flowchart of patient selection

We conducted a retrospective study on hypertensive patients who underwent both head MRI (including SWI sequences) and head CT examinations from two hospitals. Patients from Hospital 1, between February 2016 and December 2025, were randomly allocated to a training cohort and an internal validation cohort in a 7:3 proportion. Patients from Hospital 2, between January 2025 and March 2026, were assigned to the external validation cohort. This study was approved by the local ethics committees of the both hospitals. Informed consent from patients was not required. The inclusion criteria were as follows: (1) diagnosed with hypertension; (2) head CT performed within 2 weeks before or after the SWI examination. The exclusion criteria were: (1) lack of complete clinical data; (2) previous brain contusion or subarachnoid hemorrhage; (3) history of craniotomy; (4) poor image quality; (5) high likelihood of CAA per the Boston criteria [21]. In total, 345 patients from Hospital 1 were randomly allocated to a training cohort (*n* = 244) and an internal validation cohort (*n* = 101). Additionally, 113 patients from Hospital 2 were enrolled as the external validation cohort (Fig. 1).

### Clinical data collection

Clinical data were gathered from the medical systems of the institutions. The dataset comprised gender, age, hypertension duration (< 10 years or ≥ 10 years), hypertension grade, history of anticoagulant drug use, cerebral infarction, coronary heart disease, diabetes, hyperlipidemia, smoking, and drinking.

### Image acquisition

Each patient underwent MRI examinations, including SWI sequences, on one of the following MRI systems: Siemens Aera 1.5 T, Siemens MAGNETOM Vida 3.0 T, or GE Signa Voyager 1.5 T. SWI acquisition parameters were as follows: repetition time (TR) 40 ms, echo time (TE) 25 ms, slice thickness 1.8 mm with no slice interval, flip angle 30°, matrix size 320 × 320, and field of view 230 × 230 mm. The raw data were transferred to the MRI workstation for post-processing to generate SWI images.

Head CT scans were conducted with multidetector CT scanners (United Imaging 16-slice, Siemens SOMATOM Force, or Philips Brilliance 64-slice). The parameters for CT acquisition involved a tube current of 250 mA, a tube voltage of 120 kV, a matrix of 512 by 512, a field of view of 250 mm by 250 mm, and a slice thickness and interval of 5 mm each.

### Image analysis

CMBs appeared as circular areas with uniform signal loss, measuring between 2 and 10 mm in diameter, and having clear boundaries on SWI images [22, 23]. Two groups were formed from the participants based on the count of CMBs: those with > 10 CMBs and those with ≤ 10 CMBs.

Head CT features were analyzed as described below. Lacunar infarctions (LIs) were identified as round or oval hypodense (black) areas measuring 3–15 mm in diameter, located in the deep white matter, basal ganglia, pons, or cerebellum on CT scans [24]. LIs counts were graded as follows: grade 0, no LIs; grade 1, 1–4 LIs; grade 2, 5–10 LIs; and grade 3, > 10 LIs [14]. Additionally, the locations affected by LIs were categorized into three regions: cerebral hemispheres, cerebellar hemispheres, and brainstem. Based on the number of involved regions, LIs locations were graded as follows: grade 0: no LIs; grade 1: involvement of one region; grade 2: involvement of two regions; and grade 3: involvement of all three regions. Brain atrophy was assessed on a global cortical atrophy scale, with grade 0 representing “none,” grade 1 representing “mild,” grade 2 representing “moderate,” and grade 3 representing “severe” [25]. Leukoaraiosis (LA) was evaluated based on the Blennow grading scale, where scores vary from 0 to 3 [26].

SWI images were independently reviewed by two radiologists (with 12 and 13 years of experience in neuroimaging, respectively), who were blinded to the CT results. Head CT images were independently analyzed by two other radiologists (with 14 and 16 years of experience in neuroimaging, respectively), without any information about the SWI results. Interobserver agreement was assessed. Any disagreements between the two observers were mediated by a neuroradiologist with over three decades of experience.

### Statistical analysis

An intraclass correlation coefficient (ICC) model was used to assess interobserver variability for imaging features. Categorical variables were compared using the chi-squared test or Fisher’s exact test, as appropriate. The independent-sample t-test or the Mann-Whitney U test was employed to compare continuous variables when applicable. Restricted cubic splines were used to evaluate the associations between partial continuous variables and high CMBs burden. Multivariate analysis was conducted for statistically significant variables identified from the univariate analysis in the training cohort. A logistic regression model was constructed based on the selected features after multivariate analysis. Referring to the calculation formula by Ben et al. [27] (B/Bmin × 2, where B is the regression coefficient of each variable and Bmin is the minimum regression coefficient), the weighted score for each independent factor was calculated by rounding. The total score of the model was obtained by summing the scores of each independent factor, and a scoring system was then established. The DeLong test was used to compare the differences between the scoring system and the logistic regression model. The receiver operating characteristic (ROC) curve, decision curve analysis (DCA), and Hosmer-Lemeshow test were employed to evaluate the reliability and performance of the scoring system in the training, internal validation, and external validation cohorts. This study was conducted in accordance with the TRIPOD specification.

The software R version 4.1.2, SPSS 23.0, and MedCalc version 19.0 were applied for statistical analyses, with statistical significance defined as P-value < 0.05.

## Results

### Clinical and imaging features in patients

**Fig. 2:**
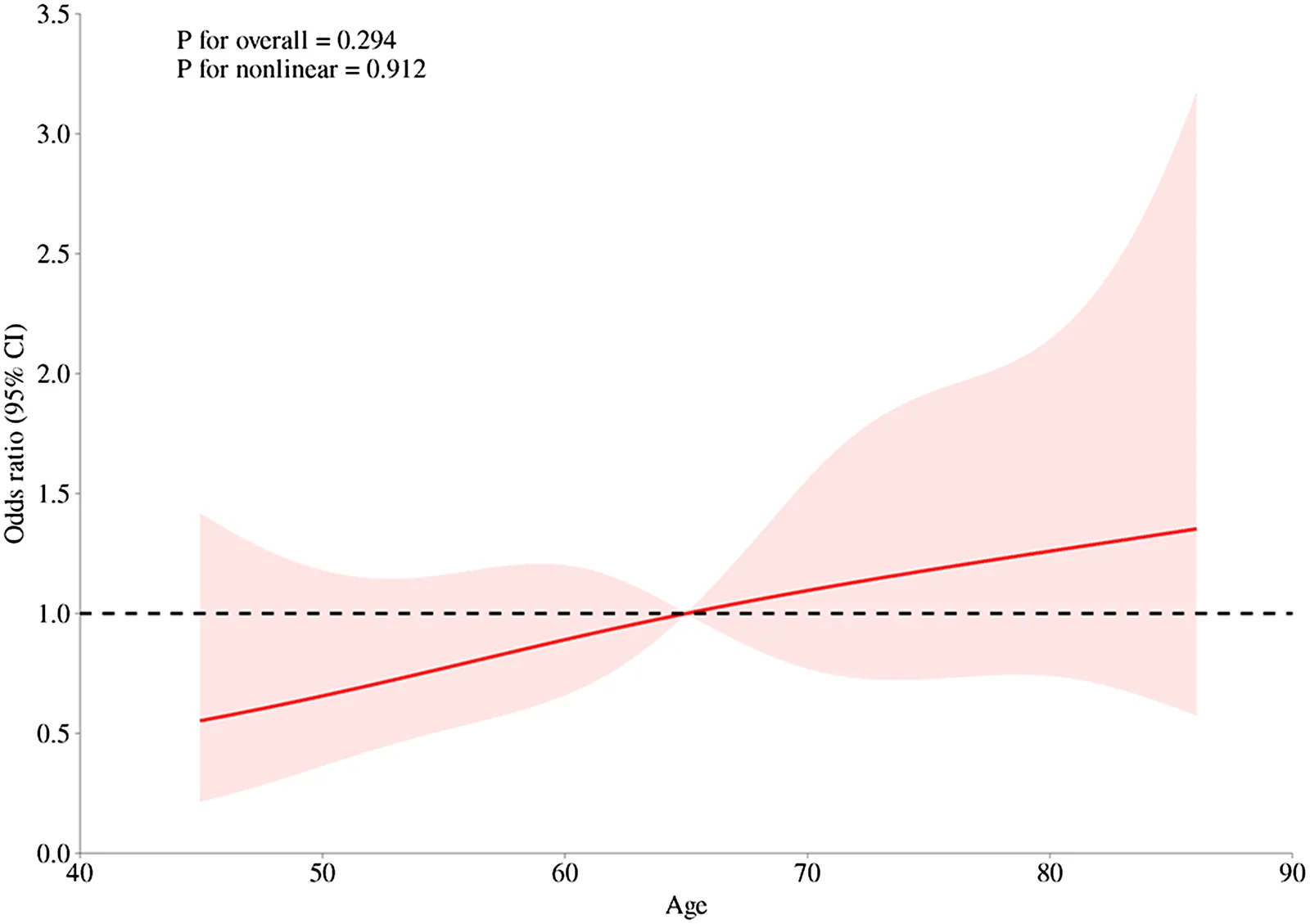
Restricted cubic spline analysis of the association between age and high CMBs burden

Differences in clinical and CT features between patients with > 10 CMBs and ≤ 10 CMBs are presented in Table 1. The LIs count grade, LIs location grade and LA grade showed statistically significant differences in the training, internal validation, and external validation cohorts (*P* < 0.05). Hypertension grade and cerebral infarction demonstrated statistically significant differences in both the training and internal validation cohorts (*P* < 0.05). Hypertension duration exhibited statistically significant differences in both the training and external validation cohorts (*P* < 0.05). A history of anticoagulant drug use and smoking history showed statistically significant differences only in the internal validation cohort (*P* < 0.05). No statistically significant differences were observed among the remaining variables (*P* > 0.05). In Fig. 2, the restricted cubic spline showed that the P for overall = 0.294 > 0.05, and the 95% confidence interval consistently crossed OR = 1, both indicating that there is no statistically significant association between age and high CMBs burden.

The ICC for > 10 CMBs was 0.969. For the grading of LIs count, LIs location, brain atrophy, and LA, the ICCs were all above 0.80, indicating good to excellent interobserver agreement.

**Table 1:** Comparison of patients in clinical and head CT features

Variable		Training cohort				*P* value	Internal validation cohort			*P* value	External validation cohort		
		> 10 CMBs (*n* = 84)		≤ 10 CMBs (*n* = 160)			> 10 CMBs (*n* = 36)	≤ 10 CMBs (*n* = 65)			> 10 CMBs (*n* = 48)	≤ 10 CMBs (*n* = 65)	*P* value
Male [n (%)]		48 (57.14)		84 (52.50)		0.489	25 (69.44)	32 (49.23)		0.050	34 (70.83)	45 (69.23)	0.854
Age (years)		67.81±12.08		64.59 ± 12.58		0.055	71.08 ± 9.19	69.55 ± 12.17		0.513	73.90 ± 10.73	73.34±11.14	0.790
Hypertension duration [n (%)]						< 0.001				0.251			0.046
< 10 years		29 (34.52)		119 (74.37)			14 (38.89)	33 (50.77)			26 (54.17)	47 (72.31)	
≥ 10 years		55 (65.48)		41 (25.63)			22 (61.11)	32 (49.23)			22 (45.83)	18 (27.69)	
Hypertension grade [n (%)]					0.002					0.009			0.356
Grade 1		10 (11.90)		51 (31.88)			10 (27.78)	36 (55.38)			6 (12.50)	15 (23.08)	
Grade 2		33 (39.29)		43 (26.87)			6 (16.67)	12 (18.46)			12 (25.00)	15 (23.08)	
Grade 3		41 (48.81)		66 (41.25)			20 (55.55)	17 (26.15)			30 (62.50)	35 (53.85)	
Anticoagulant drugs [n (%)]		18 (21.43)		24 (15.00)	0.206		15 (41.67)	12 (18.46)		0.012	7 (14.58)	9 (13.85)	0.912
Cerebral infarction [n (%)]		40 (47.62)		43 (26.87)	0.001		13 (36.11)	7 (10.77)		0.002	20 (41.67)	16 (24.62)	0.054
Coronary heart disease [n (%)]		10 (11.90)		22 (13.75)	0.685		1 (2.78)	9 (13.85)		0.151	11 (22.92)	9 (13.85)	0.212
Diabetes [n (%)]		16 (19.05)		28 (17.50)	0.765		6 (16.67)	13 (20.00)		0.681	10 (20.83)	11 (16.92)	0.597
Hyperlipidemia [n (%)]		5 (5.95)		15 (9.37)	0.354		3 (8.33)	2 (3.07)		0.243	2 (4.17)	6 (9.23)	0.505
Smoking [n (%)]		29 (34.52)		52 (32.50)	0.750		17 (47.22)	15 (23.07)		0.012	19 (39.58)	28 (43.08)	0.710
Drinking [n (%)]		28 (33.33)		44 (27.50)	0.342		12 (33.33)	12 (18.46)		0.150	12 (25.00)	17 (26.15)	0.890
LIs count [n (%)]					< 0.001				< 0.001				< 0.001
Grade 0–1		30 (35.71)		143 (89.38)			7 (19.44)	54 (83.07)			5 (10.42)	33 (50.77)	
Grade 2–3		54 (64.29)		17 (10.62)			29 (80.56)	11 (16.92)			43 (89.58)	32 (49.23)	
LIs location [n (%)]					< 0.001				< 0.001				< 0.001
Grade 0–1		28 (33.33)		134 (83.75)			13 (36.11)	51 (78.46)			20 (41.67)	53 (81.54)	
Grade 2–3		56 (66.67)		26 (16.25)			23 (63.89)	14 (21.54)			28 (58.33)	12 (18.46)	
Brain atrophy [n (%)]					0.140					0.113			0.460
Grade 0–1		59 (70.24)		126 (78.75)			23 (63.89)	51 (78.46)			21 (43.75)	33 (50.77)	
Grade 2–3		25 (29.76)		34 (21.25)			13 (36.11)	14 (21.54)			27 (56.25)	32 (49.23)	
LA grade [n (%)]					< 0.001				< 0.001				< 0.001
Grade 0–1		11 (13.09)		111 (69.37)			6 (16.67)	40 (61.54)			15 (31.25)	47 (72.31)	
Grade 2–3		73 (86.90)		49 (30.63)			30 (83.33)	25 (38.46)			33 (68.75)	18 (27.69)	

CMBs, cerebral microbleeds; LIs, lacunar infarcts; LA, leukoaraiosis

### Development of a logistic regression model

In the univariate analysis, hypertension duration, hypertension grade, cerebral infarction, LIs count, LIs location, and LA grade were significantly different between the > 10 CMBs and ≤ 10 CMBs groups in the training cohort. In the multivariate logistic analysis, hypertension duration (odds ratio [OR]: 2.631, 95% confidence interval [CI]: 1.245–5.560, *p* = 0.011), LIs count (OR: 4.732, 95% CI: 2.072–10.807, *p* < 0.001), LIs location (OR: 3.649, 95% CI: 1.631–8.164, *p* = 0.002), and LA grade (OR: 7.202, 95% CI: 3.161–16.409, *p* < 0.001) were identified as independent factors predicting > 10 CMBs (Table 2). The variance inflation factors (VIFs) for these independent risk factors were all under 2. The logistic regression model had an area under the curve (AUC) of 0.899 (95% CI: 0.859–0.939, *P* < 0.001), and the Hosmer-Lemeshow test confirmed good calibration of the model (*P* = 0.402, > 0.05).

### Development of a scoring system

Based on the B values shown in Table 2, weighted scores were allocated to each feature as follows: LIs count (grade 2–3), 3 points; LIs location (grade 2–3), 3 points; LA (grade 2–3), 4 points; and hypertension duration (≥ 10 years), 2 points. A scoring system ranging from 0 to 12 was ultimately developed by adding up the individual scores linked to the relevant variables.

**Table 2:** Univariate and multivariate logistic regression analyses of clinical and CT features

Variable	OR Univariate analysis *P*			B OR Multivariate analysis *P*				VIF
		95% CI				95% CI		
Male	1.206	0.709–2.054	0.489					
Age	0.130	0.999–1.044	0.056					
Hypertension duration	5.505	3.104–9.763	< 0.001	0.967	2.631	1.245–5.560	0.011	1.197
Hypertension grade	1.549	1.100-2.182	0.012	0.413	1.511	0.911–2.507	0.110	1.027
Anticoagulant drugs	1.545	0.784–3.045	0.208					
Cerebral infarction	2.472	1.423–4.299	0.001	-0.093	0.911	0.416–1.996	0.817	1.123
Coronary heart disease	0.848	0.381–1.885	0.685					
Diabetes	1.109	0.562–2.190	0.765					
Hyperlipidemia	0.612	0.241–1.746	0.358					
Smoking	1.095	0.627–1.914	0.750					
Drinking	1.318	0.745–2.334	0.343					
LIs count	15.141	7.730-29.658	< 0.001	1.554	4.732	2.072–10.807	< 0.001	1.618
LIs location	10.308	5.555–19.127	< 0.001	1.294	3.649	1.631–8.164	0.002	1.542
Brain atrophy	1.570	0.860–2.867	0.142					
LA grade	15.033	7.336–30.809	< 0.001	1.974	7.202	3.161–16.409	< 0.001	1.345

LIs, lacunar infarcts; LA, leukoaraiosis; VIF, variance inflation factor

### Performance and clinical utility of the scoring system

**Fig. 3:**
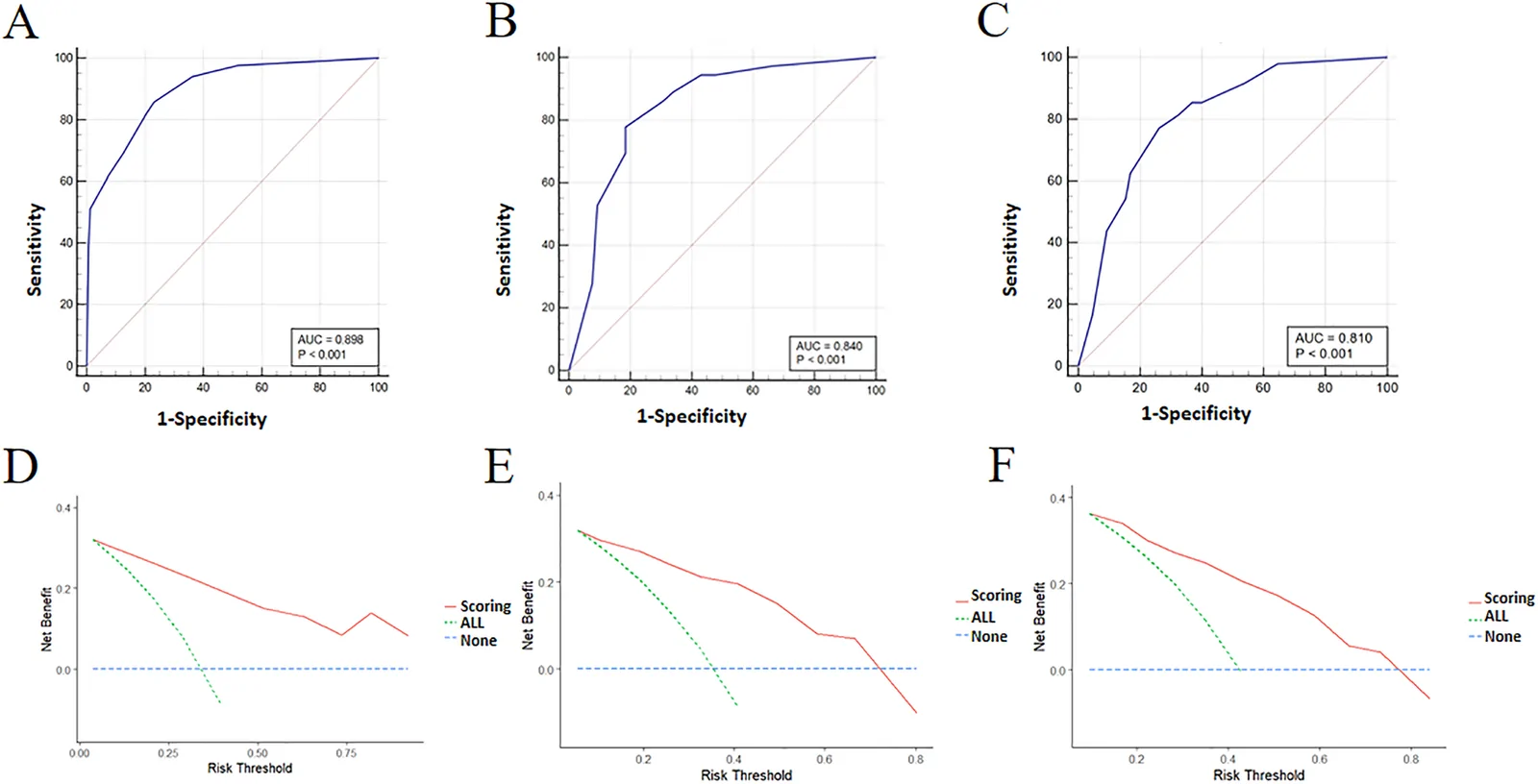
Performance and clinical utility of the scoring system. ROC curves of the scoring system for predicting > 10 CMBs in the training (**A**), internal validation (**B**), and external validation cohorts (**C**). The AUC values for the training, internal validation, and external validation cohorts were 0.898 (95% CI: 0.857–0.938), 0.840 (95% CI: 0.760–0.920), and 0.810 (95% CI: 0.725–0.877), respectively. DCA for the scoring system in the training (**D**), internal validation (**E**) and external validation cohorts (**F**)

The ROCs of the scoring system are shown in Fig. 3A, B, and C. In the training cohort, the AUC of the scoring system was 0.898 (95% CI: 0.857–0.938). At a cutoff score of 4.5 points, the sensitivity was 85.7%, and the specificity was 76.9%. The result of the Hosmer-Lemeshow test showed a P-value of 0.391 (> 0.05). In the internal validation cohort, the AUC was 0.840 (95% CI: 0.760–0.920), with a sensitivity of 77.8% and a specificity of 81.5%. In the external validation cohort, the AUC was 0.810 (95% CI: 0.725–0.877), with a sensitivity of 77.1% and a specificity of 73.8%. The Hosmer-Lemeshow test results for the internal and external validation cohorts showed P-values of 0.481 and 0.684, respectively.

Additionally, the DCA of the scoring system (red line) was higher than both the intervention-all strategy (green line) and the intervention-none strategy (blue line) across a broad range of threshold probabilities in the training, internal validation, and external validation cohorts (Fig. 3D, E, F).

**Fig. 4:**
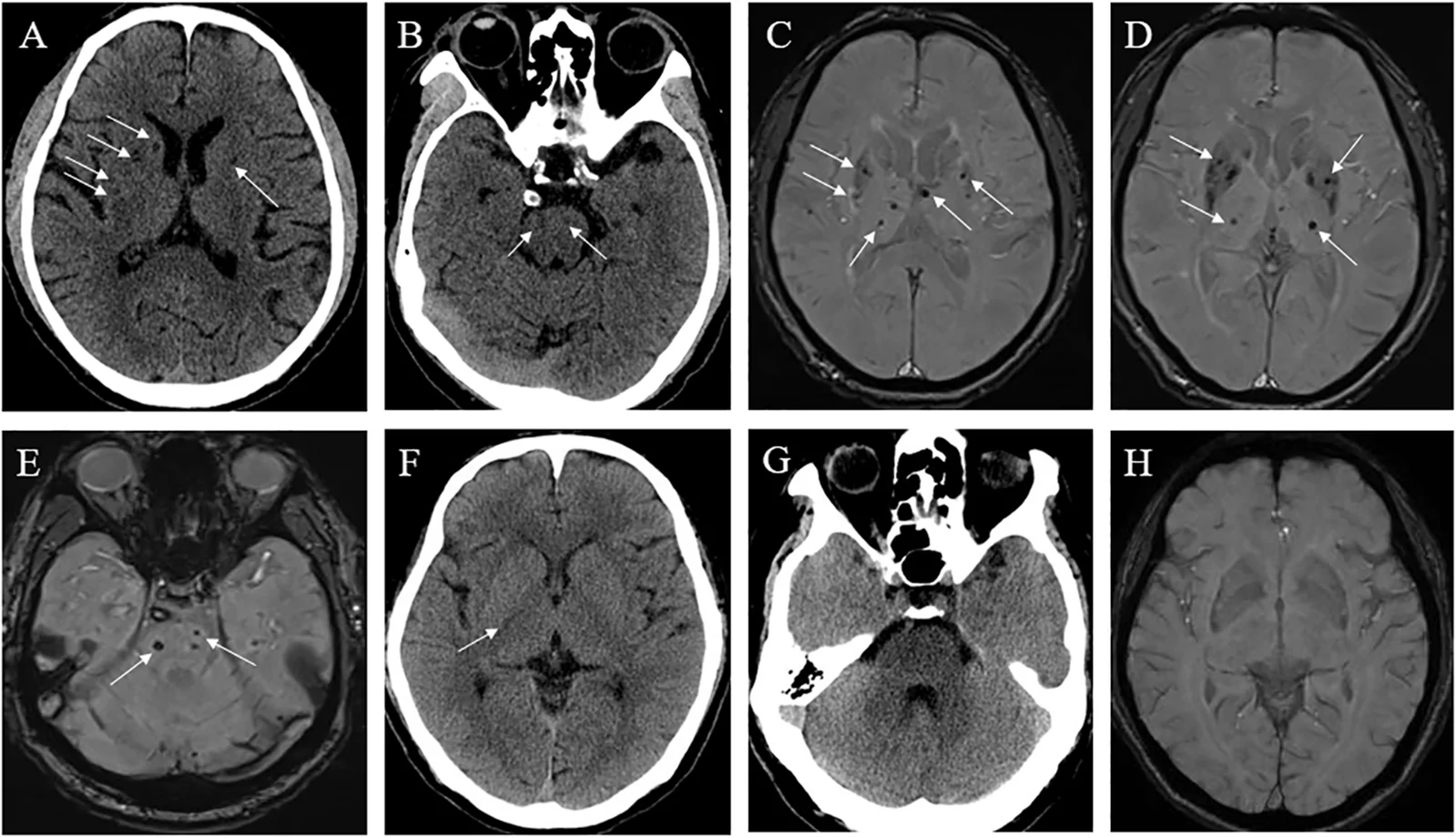
The scoring system was used for predicting > 10 CMBs in the hypertensive population. (**A-E**). Case 1: a male with hypertension for 30 years. Head CT demonstrated multiple LIs (> 5) (white arrows) located in the bilateral basal ganglia and brainstem regions. No signs of LA were observed (**A-B**). SWI revealed multiple CMBs (> 10) (white arrows) (**C-E**). The patient scored 2 points for hypertension duration and 3 points each for the grade of count and location of LIs, resulting in a total score of 8 (ranging 5–12). This score, which suggested > 10 CMBs, was ultimately consistent with the SWI findings (**F-H**). Case 2: a male with hypertension for 8 years. Head CT showed only one LIs in the right basal ganglia (**F-G**). SWI revealed no definite CMBs. The patient’s total score was 0 (ranging 0–1), in accordance with the SWI results (**H**)

The scoring system was further categorized into three score ranges: 0–1 points, 2–4 points, and 5–12 points. As the score increased, the incidence of > 10 CMBs progressively rose across the score ranges (Table 3). In the training cohort, the incidences of > 10 CMBs in the respective score ranges were 2.53% (2/79), 17.85% (10/56), and 66.05% (72/109). In the internal validation cohort, the corresponding incidences were 4.34% (1/23), 12.50% (3/24), and 59.26% (32/54). In the external validation cohort, the corresponding incidences were 4.17% (1/24), 25.00% (6/24), and 63.07% (41/65). When applied to practical cases, the scoring system demonstrated strong performance (Fig. 4).

**Table 3:** Diagnostic probability of > 10 CMBs in different score ranges

Score range (points)	> 10 CMBs (*n*)				All hypertension patients (*n*)				Probability of >10 CMBs		
	Training cohort	Internal validation cohort	External validation cohort		Training cohort	Internal validation cohort	External validation cohort		Training cohort	Internal validation cohort	External validation cohort
0–1	2	1	1		79	23	24		2.53%	4.34%	4.17%
2–4	10	3	6		56	24	24		17.85%	12.5%	25.00%
5–12	72	32	41		109	54	65		66.05%	59.26%	63.07%

CMBs, cerebral microbleeds

### Sample size estimation

Initially, we attempted to calculate the sample size based on standard rule-of-thumb criteria, N > EPV × k / P [28]. Unfortunately, despite our best efforts, we were unable to find data on the incidence of high CMBs burden in hypertensive patients from the literatures. Finally, we calculated the required sample size based on the incidence of high CMBs burden in our First Medical Center data (training set and internal validation set), which was approximately 34.8%. With k = 15 (number of independent variables) and EPV set to a minimum of 10, the estimated required sample size was 431. Our total sample size was 458, which exceeds 431 and meets the minimum sample size requirement.

## Discussion

We successfully developed and validated a scoring system utilizing clinical and head CT features to predict high CMBs burden in hypertensive patients. The model demonstrated strong performance across the training (AUC: 0.898), internal validation (AUC: 0.840), and external validation (AUC: 0.810) cohorts, suggesting its potential utility in clinical practice.

The scoring system included one clinical feature and three CT features: hypertension duration, LIs count grade, LIs location grade, and LA grade. A hypertension duration of ≥ 10 years was strongly associated with high CMBs burden, reinforcing that cumulative exposure to elevated blood pressure exacerbates microvascular damage, consistent with a dose–response relationship reported previously [29, 30]. Although hypertension grade and cerebral infarction were significant in univariate analyses, they did not remain independent predictors in multivariate logistic regression, possibly due to weaker effects mediated by other variables. On CT images, LIs and LA were key predictors, serving as imaging markers of chronic cerebral small vessel disease (CSVD) [31, 32]. LIs represent small, healed infarcts in deep brain structures, while LA reflects ischemic white matter damage. Their strong predictive value reinforces that different CSVD subtypes often coexist as a global cerebral microangiopathy [33, 34]. Our results were consistent with studies showing that LIs and LA on neuroimaging are significant risk factors for CMBs on MRI [35, 36]. Notably, this study is the first to include a CT‑based grading of LIs location as a significant feature. All four variables had VIFs below 2, indicating no multicollinearity.

In the training cohort, the logistic regression model achieved an AUC of 0.899, while the scoring system yielded an AUC of 0.898 (Delong test, *P* > 0.05), indicating that the scoring system preserved the core predictive ability and is clinically applicable. In the internal and external validation cohorts, the AUCs were 0.840 and 0.810, respectively, demonstrating strong clinical performance and stability. The Hosmer‑Lemeshow test yielded *P* > 0.05 across all cohorts, indicating satisfactory calibration. DCA showed that the model offered significantly greater net benefits within clinically relevant thresholds compared to ‘treat‑all’ and ‘treat‑none’ strategies. Furthermore, a higher score was associated with a higher incidence of > 10 CMBs.

Compared with a previous model by Li et al. [14], which predicted high CMBs burden (> 10 CMBs) in AIS patients using three CT features (LA grade, LIs count grade, brain atrophy grade) and one clinical feature (hypertension), our model focuses specifically on the hypertensive population and benefits those with either acute ischemic stroke or atrial fibrillation. We incorporated a broader range of features and, for the first time, identified the predictive value of LIs location grade. The two CT features ultimately selected in our model (LIs count grade and LA grade) are consistent with Li’s model, highlighting their robust value. However, brain atrophy was not identified in our model, possibly due to differences in study populations leading to bias. Our model’s AUC in the training cohort (0.898) is slightly higher than that of Li’s model (AUC: 0.894), but the internal and external validation AUCs (0.840 and 0.810) are lower than that of Li’s model (AUCs: 0.902 and 0.914), suggesting that the higher complexity of our model may have introduced some overfitting.

Based on our model, for AIS patients with a score of 0–4, direct thrombolysis can be performed if no other contraindications exist. For patients with a score of 5–12, if the time window permits, MRI is recommended to avoid major bleeding risk from blind thrombolysis. For those with a score of 5–12 who cannot undergo MRI due to time constraints, especially those with a score ≥ 9, individualized decision‑making is advised. For patients with atrial fibrillation and a score of 5–12, we recommend using novel oral anticoagulants instead of conventional agents.

This study has several limitations. First, the sample size was not very large, and we plan to expand it in the future. Second, all variables (e.g., hypertension duration, LIs, LA) were binary, which may limit predictive precision; future studies will incorporate quantitative or ordinal variables. Third, only recent blood pressure control was recorded; we plan to collaborate with cardiology specialists to identify long‑term control indicators. Fourth, for diseases prone to CMBs such as CAA and cerebral autosomal dominant arteriopathy with subcortical infarcts and leukoencephalopathy (despite low incidence), our model is not applicable. How to accurately rule out these conditions before brain MRI in hypertensive patients without a confirmed history remains a key focus. Fifth, given the urgency of model application, we did not include additional clinical/laboratory features (e.g., blood pressure variability, homocysteine); these will be added in the future to improve the model’s performance. Sixth, different CT scanners and protocols may affect predictive performance; we recommend using images acquired with standardized parameters to minimize errors. Seventh, our model is still in the preliminary development stage; therefore, the clinical pathway we suggest may differ from real-world clinical practice. In the future, we will conduct further multicenter studies to improve the model’s performance and stability. When the model matures, we recommend integrating it into the electronic health records. Eighth, as this was a retrospective study, the data may have been subject to selection bias. Future efforts should include a prospective study to address this limitation. Ninth, restricted cubic splines are applicable for analyzing the association between continuous variable parameters and the dependent variable. In this study, for some patients with a history of hypertension exceeding 10 years, the specific duration was not precisely documented in the medical system; instead, it was recorded using vague terms such as “more than 10 years,” “more than 20 years,” or “more than 30 years.” Similarly, for some patients with more than 10 LIs, the exact number of lesions was not recorded in detail. These limitations precluded the application of this statistical method to these variables. Consequently, only age was ultimately included in the restricted cubic spline analysis.

## Conclusion

In summary, we developed and validated a scoring system utilizing clinical and head CT features to predict high CMBs burden in hypertensive patients. Four key predictors were incorporated into the scoring system. The model exhibited strong performance across the training cohort, as well as both internal and external validation cohorts, indicating its potential for guiding clinical decision-making.

## Data Availability

According to the restrictions of the hospital ethics committees and patient privacy protection policies, our datasets are not permitted to be publicly accessed. The datasets used or analyzed during the current study are available from the corresponding author on reasonable request.

## Abbreviations

CMBs: Cerebral microbleeds
ROC: Receiver operating characteristic
DCA: Decision curve analysis
AUC: Area under the curve
CI: Confidence interval
GRE: Gradient-recalled echo
SWI: Susceptibility-weighted imaging
CAA: Cerebral amyloid angiopathy
sICH: Symptomatic intracranial haemorrhage
MRI: Magnetic resonance imaging
CT: Computed tomography
AIS: Acute ischemic stroke
TR: Repetition time
TE: Echo time
LIs: Lacunar infarcts
LA: LEukoaraiosis
ICC: Intraclass correlation coefficient
OR: Odds ratio
VIFs: Variance inflation factors
CSVD: Chronic cerebral small vessel disease
